# Comparison of the multiples of the median of serum anti‐müllerian hormone and pregnancy outcomes in patients with gestational trophoblastic disease: A case–control study

**DOI:** 10.1002/cam4.7134

**Published:** 2024-03-28

**Authors:** Theodora Hei Tung Lai, Lesley Suk Kwan Lau, Siew Fei Ngu, Man Yee Mandy Chu, Karen Kar Loen Chan, Ernest Hung Yu Ng, Hextan Yuen Sheung Ngan, Raymond Hang Wun Li, Ka Yu Tse

**Affiliations:** ^1^ Department of Obstetrics and Gynaecology Queen Mary Hospital Hong Kong China; ^2^ Division of Gynaecological Oncology, Department of Obstetrics and Gynaecology, School of Clinical Medicine Hong Kong China; ^3^ Division of Reproductive Medicine, Department of Obstetrics and Gynaecology, School of Clinical Medicine The University of Hong Kong Hong Kong China

**Keywords:** Anti‐müllerian hormone, chemotherapy, gestational trophoblastic neoplasia, molar pregnancy, ovarian reserve

## Abstract

**Introduction:**

Chemotherapy is crucial in treating gestational trophoblastic neoplasia (GTN), but its impact on gonadotoxicity is unclear.

**Materials and Methods:**

This case–control study included 57 GTN patients and 19 age‐matched patients with molar pregnancies (MP) in 2012–2018. Multiples of the median (MoM) of the serum AMH levels were compared between the two groups, and between patients using single‐agent and combination chemotherapy, at baseline, 6, 12, and 24 months after treatment. Their pregnancy outcomes were also compared.

**Results:**

There was no significant difference in the MoM of serum AMH between GTN and MP groups at all time points. Single‐agent chemotherapy did not adversely affect the MoM. However, those receiving combination chemotherapy had lower MoM than those receiving single‐agent chemotherapy at all time points. The trend of decline from the baseline was marginally significant in patients with combination chemotherapy, but the drop was only significant at 12 months (*Z* = −2.69, *p* = 0.007) but not at 24 months (*Z* = −1.90; *p* = 0.058). Multivariable analysis revealed that combination chemotherapy did not affect the MoM. There was no significant difference in the 4‐year pregnancy rate and the livebirth rate between the single‐agent and combination groups who attempting pregnancy, but it took 1 year longer to achieve the first pregnancy in the combination group compared to the single‐agent group (2.88 vs. 1.88 years).

**Conclusion:**

This study showed combination chemotherapy led to a decreasing trend of MoM of serum AMH especially at 12 months after treatment, but the drop became static at 24 months. Although pregnancy is achievable, thorough counseling is still needed in this group especially those wish to achieve pregnancy 1–2 years after treatment or with other risk factors.

## Graphical abstract

Our study was also the first to use MoM of serum AMH to examine the effect of chemotherapy in GTD. We showed a trend of decrease in those receiving combination chemotherapy, but it became static between 12 and 24 months and the live birth rate was not jeopardized by the chemotherapy.

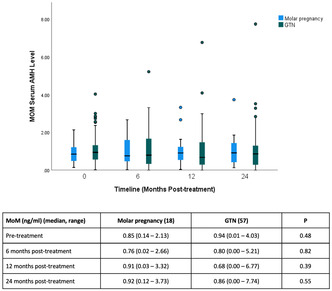

## 1 INTRODUCTION

Gestational trophoblastic disease (GTD) is a rare pregnancy‐related condition consisting of benign and malignant subtypes. While most patients with molar pregnancies (MP) do not require chemotherapy but only monitoring of serum human chorionic gonadotrophin (hCG) levels, those with gestational trophoblastic neoplasia (GTN) require either single‐agent or combination chemotherapy. The median age at diagnosis of GTN was 24.5–32.5 years old.^1^, ^2^, ^3^, ^4^ GTN is highly chemo‐sensitive and disease remission can be achieved in more than 90% of patients even at advanced stage.^5^ Given the high rates of survival, it is important to address the potential long‐term impact of chemotherapy on these young patients, especially their future fertility. It had been shown that one in 10 young women with breast cancer would forgo the chances of cure from malignancy in exchange for maintaining their childbearing potential.^6^ This highlights the importance of fertility in these young survivors.

In theory, chemotherapy could lead to a loss of ovarian follicles and hence an accelerated decline in fertility potential.^7^ The magnitude of gonadotoxicity and the risk of premature ovarian insufficiency depends on many factors such as the age at diagnosis, the type and location of malignancy, and the type and duration of the medical treatments. One Japanese study described that patients with GTN who received chemotherapy would have a greater decline in ovarian reserve than those with MP not requiring chemotherapy.^8^ However, a livebirth rate of up to 70% within 1 year of chemotherapy had also been reported in the literature.^7^

With the conflicting results in the literature, we aimed to characterize the effects of chemotherapy on the ovarian reserve between patients with GTN who received chemotherapy and patients with MP without chemotherapy, and also between patients using single‐agent and combination chemotherapy. As the depletion of ovarian reserve after chemotherapy may not render the woman permanently amenorrhoeic, menstrual pattern is not a reliable marker of the remaining ovarian reserve following chemotherapy.^9^, ^10^ In this study, serum anti‐Müllerian hormone (AMH) was used as a surrogate marker for ovarian reserve that had been used as an indicator of follicular depletion and recovery in cancer patients during and after chemotherapy.^11^

## 2 MATERIALS AND METHODS

### 2.1 Patient selection

Patients were identified from the gynecological oncology database of Queen Mary Hospital, a university‐affiliated tertiary hospital in Hong Kong, between Jan 2012 and Nov 2018. As a referral center, the hospital managed 102 new GTN patients over the same period. Their diagnoses and stages were confirmed again by reviewing their medical records. All patients aged between 20 and 44 years diagnosed with GTN requiring chemotherapy were included. This age range was chosen to match the age range of a model constructed by our group so that the age‐specific multiples of the median (MoM) could be generated.^12^ Exclusion criteria included patients who aged 45 and above, those who received previous chemotherapy or treatment for other malignancies, and those who had used exogenous hormones within 3 months prior to the collection of baseline blood samples. Patients who did not have serum samples at all the predefined time points (pretreatment, 6 months, 12 months, and 24 months) were also excluded. Patients with GTN were matched with patients who had MP without the need for chemotherapy in 3:1 ratio. They were identified and selected at random from the database and were matched for the age group within 5 years as the patients with GTN (e.g., 20–24, 25–29 till 40–44). This study was approved by the Institutional Review Board of The University of Hong Kong‐Hospital Authority (Hong Kong West Cluster) (reference number: UW 11–298), and it was conducted in accordance with the Declaration of Helsinki.

### 2.2 Definitions

GTD included both MP and GTN. GTN incorporates the histopathological entities of malignant invasive mole, choriocarcinoma, placental site trophoblastic tumor (PSTT), and epithelioid trophoblastic tumor (ETT), and was diagnosed by a plateau of hCG with four weekly measurements over 3 weeks or a rise of hCG with three weekly measurements over 2 weeks after any pregnancy.^13^ The International Federation of Gynecology and Obstetrics (FIGO) staging system and World Health Organization (WHO) scoring systems were used, where a WHO score of 7 or higher was considered the high‐risk group. Patients with high‐risk GTN, including FIGO stage III–IV and/or score of 7 or above received combination chemotherapy, either EMA‐CO (etoposide, methotrexate, actinomycin‐D, cyclophosphamide, and vincristine with folinic acid rescue) or CHAMOC (cyclophosphamide, hydroxyurea, actinomycin‐D, methotrexate with folinic acid, vincristine). Patients with low‐risk GTN received single‐agent chemotherapy only, mostly methotrexate, and occasionally actinomycin‐D as a second‐line alternative. Consolidation with three more cycles of chemotherapy was usually given, except in low‐risk GTN patients who had satisfactory fall of hCG after 1 cycle of chemotherapy, or in patients who could not tolerate the chemotherapy. Patients who initially received single agent but subsequently switched to another agent or combination chemotherapy were categorized as the combination group in this study.

### 2.3 Study procedures

Eligible patients with MP and GTN were longitudinally followed up at our designated clinic. All patients underwent serial blood tests for hCG monitoring, and their remaining sera samples were archived at −80°C in the Hormone Laboratory of our Department. Archived serum samples at pretreatment as well as at 6 months, 12 months, and 24 months after treatment were retrieved, and AMH was measured using the Access AMH assay (Beckman‐Coulter Inc, Marseille cedex, France). Various cutoff values of serum AMH have been suggested. However, these could vary with different AMH assays and the age of different study cohorts. Given the age‐dependent nature of AMH, and that the GTN and control group might not be completely age‐matched, the use of MoM could potentially circumvent all these problems. Therefore, all the serum AMH levels in this study were expressed as MoM based on the age‐specific AMH reference ranges for healthy Chinese women as previously described.^12^

Patients' demographic and clinical characteristics such as the age, gravidity, parity, body mass index (BMI) and chemotherapy regimen, were collected. Their pregnancy outcomes were collected up to Mar 2023.

### 2.4 Statistical consideration

The primary endpoint was the difference in MoM of the serum AMH levels at different time points between patients with GTN and MP. Secondary endpoints included the same difference between patients using single‐agent and combination chemotherapy in GTN patients, as well as the pregnancy and livebirth rates in different groups.

Data were reported as counts (percentages) for categorical variables, mean (+/− standard deviation (SD)) for normally distributed continuous variables, or median (interquartile or entire range) for non‐normally distributed continuous variables. Chi‐square test or Fisher's exact test were used to compare categorical variables between groups. Student's *t* test or Mann–Whitney *U* test was used to compare continuous variables where appropriate. Friedman's test and Wilcoxon signed rank test were used to compare repeated measures of continuous variables within subjects in the same group and across different groups, respectively. Multivariate analysis was performed by binomial logistic regression. *p* values of <0.05 were considered clinically significant. Statistical analysis was performed using SPSS Statistics 28.0 (IBM Corporation, Armonk, NY, USA).

## 3 RESULTS

### 3.1 Demographic factors

A total of 76 patients diagnosed with GTD during the study period were included. Among them, 57 had GTN, including 49 with post‐molar GTN and 8 with choriocarcinoma, who received chemotherapy. Nineteen patients with MP, including eight partial moles and 11 complete moles served as the MP group. There were no significant differences in most of the demographic factors between the GTN and MP groups (Table 1), except the gravidity (*p* = 0.01) and parity (*p* = 0.03), which were not clinically meaningful due to the small difference in the actual numbers of pregnancies. The methods of contraception before and after treatment, such as barrier (regular and irregular use) and contraceptive pills, were also shown.

**TABLE 1:** Demographic factors of patients with molar pregnancy and gestational trophoblastic neoplasia.

	Molar pregnancy (*n* = 19)	GTN (*n* = 57)	*p*
Age at diagnosis, years (median, 25th–75th percentiles)	34.7 (31.4–38.8)	35.1 (31.6–40.1)	0.46
Body mass index, kg/m^2^ (median, 25th–75th percentiles)	21.6 (19.8–24.2)	21.6 (20.1–24.9)	0.52
Previous pelvic surgeries			0.37
Yes	3 (15.8)	16 (28.1)	
No	16 (84.2)	41 (71.9)	
Menstrual regularity pretreatment (*N*, %)			0.59
Regular	17 (89.5)	46 (80.7)	
Oligomenorrhea	2 (10.5)	9 (15.8)	
Amenorrhea	0 (0)	2 (3.5)	
History of subfertility (*N*, %)	1 (5.3)	1 (1.8)	0.44
Gravidity at diagnosis (median, 25th–75th percentiles)	2 (1–2)	3 (2–4)	0.01
Parity at diagnosis (median, 25th–75th percentiles)	0 (0–1)	1 (0–1)	0.03
Number of previous curettage/suction evacuation (median, 25th–75th percentiles)	0 (0–1)	0 (0–1)	0.34
Pretreatment contraception method (*N*, %)			0.30
None	11 (57.9)	43 (75.4)	
Barrier	8 (42.1)	12 (21.1)	
Combined hormonal contraception	0 (0)	1 (1.8)	
Progestogen only contraception	0 (0)	1 (1.8)	
Intra‐uterine contraceptive device	0 (0)	0 (0)	
Sterilization	0 (0)	0 (0)	
Post‐treatment contraception method (N, %)			0.25
None	1 (5.3)	2 (3.5)	
Barrier	17 (89.5)	52 (91.2)	
Combined hormonal contraception	0 (0)	3 (5.3)	
Progestogen only contraception	0 (0)	0 (0)	
Intra‐uterine contraceptive device	1 (5.3)	0 (0)	
Sterilization	0 (0)	0 (0)	

The clinical features of GTN patients were illustrated in Table 2. Nearly 80% of the GTN were preceded by MP. It was noteworthy that 19.3% of patients in our cohort were diagnosed at stage III/IV and 17.5% had high WHO risk scores ranging from 7 to 14. Among all the GTN patients, 35.1% required combination chemotherapy, and 95% of them required more than 3 cycles.

**TABLE 2:** Clinical features of the 57 patients with gestational trophoblastic neoplasia.

	Number (%)
Antecedent pregnancy (*N*, %)	
Miscarriage/abortion/ectopic pregnancy	5 (8.8)
Partial mole	3 (5.3)
Complete mole	41 (71.9)
Normal pregnancy	8 (14.0)
Stage (*N*, %)	
I	46 (80.7)
III	7 (12.3)
IV	4 (7.0)
Risk scoring classification (*N*, %)	
Low risk (WHO score <7)	47 (82.5)
High risk (WHO score > = 7)	10 (17.5)
World Health Organization score (median, 25th–75th percentiles)	3 (1–3.5)
Chemotherapy regimen (*N*, %) (total = 57)	
Single agent chemotherapy	37 (64.9)
Combination chemotherapy	20 (35.1)
Single agent chemotherapy (*N*, %) (total = 37)	
≤3 cycles	18 (48.6)
>3 cycles	19 (51.4)
Combination chemotherapy (*N*, %) (total = 20)	
≤3 cycles	1 (5.0)
>3 cycles	19 (95.0)

### 3.2 Changes in MoM of serum AMH between groups

**FIGURE 1:**
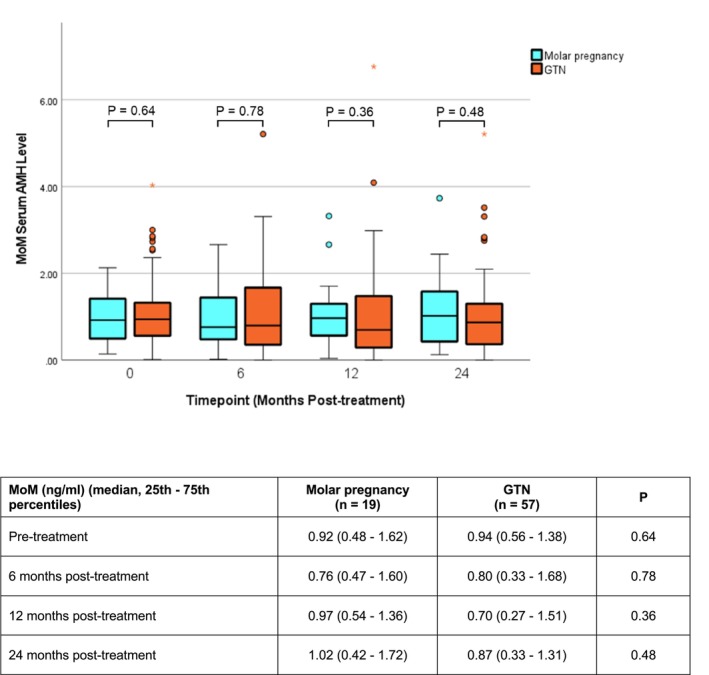
Boxplot comparing the serial MoM in molar pregnancy and GTN patients.

The serial changes in the MoM of serum AMH between the GTN and MP groups were illustrated in Figure 1. When comparing between the GTN and MP groups, there was no significant difference in the MoM of serum AMH at baseline and all the time points.

**FIGURE 2:**
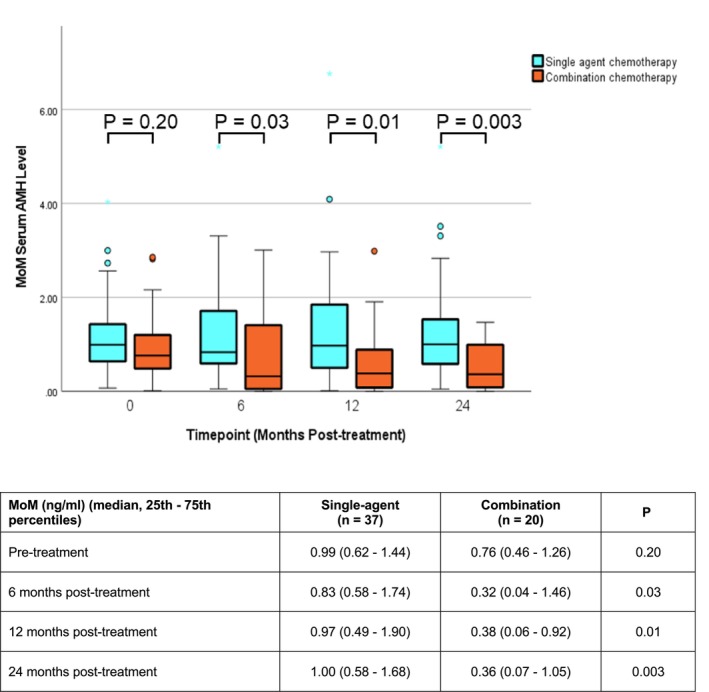
Boxplot comparing the serial MoM in patients with GTN receiving single‐agent or combination chemotherapy.

When comparing the MoM of serum AMH between those receiving single‐agent chemotherapy and combination chemotherapy at different time points, there was no significant difference between the two groups at baseline, though the level was slightly higher in the single‐agent group. However, the difference became significant at 6 months (*Z* = −2.12; *p* = 0.03), 12 months (*Z* = −2.47; *p* = 0.01), and 24 months (*Z* = −2.96, *p* = 0.003) after treatment, respectively (Figure 2).

### 3.3 Changes in MoM of serum AMH within individual groups

Next, we evaluated the internal change in the MoM of serum AMH within individual groups. Within the MP group alone, the trend of decline in the MoM over time from the baseline was not significant (X^2^(3) = 1.55, *p* = 0.67). In contrast, the decreasing trend was significant in the GTN group (X^2^(3) = 8.54; *p* = 0.036). Post hoc analysis revealed that the difference was only significant at 12 months after treatment compared to the baseline (Z = −2.79, *p* = 0.005), and the difference became insignificant after 24 months (Z = −0.73, *p* = 0.468) (Table S1).

Similarly, no significant decreasing trend in the MoM was observed in patents receiving single‐agent chemotherapy (X^2^(3) = 4.40; *p* = 0.22). On the contrary, for those receiving combination chemotherapy, a marginally significant decreasing trend was observed (X^2^(3) = 8.04; *p* = 0.05). Specifically in the combination chemotherapy group, while there was no significant change in MoM of serum AMH at 6 months from the baseline (*Z* = −1.29, *p* = 0.20), a significant drop was observed at 12 months (*Z* = −2.69, *p* = 0.007). However, the drop became less significant at 24 months (Z = −1.90; *p* = 0.058) after treatment (Table S2), and the MoM levels were actually similar at 12 (0.38; 95% CI 0.06–0.92) and 24 months (0.36; 95% CI 0.07–1.05) (Figure 2).

### 3.4 Multivariable analysis in GTN patients

The median percentage change of MoM of serum AMH in the 57 GTN patients was 10% at 24 months after treatment (Figure 1). We subsequently divided the GTN patients into two groups using this value as the cutoff, and correlated the MoM groups with different clinical parameters (Table 3). Multivariable analysis was performed to evaluate the impact of selected risk factors on serum AMH, including age at baseline, WHO risk score, chemotherapy (single‐agent or combination), and the number of cycles of chemotherapy (Table 4). None of these, including the use of combination chemotherapy, was found to be significant.

**TABLE 3:** Comparison of clinical parameters of patients with multiple of median of serum AMH level 24 months after chemotherapy in patients with gestational trophoblastic neoplasia.

Median percentage change of MoM	24 Months post‐chemotherapy		
	<10%	≥10%	*p*
Body mass index, kg/m^2^ (median, 25th–75th percentiles)	22.5 (19.3–25.3)	20.9 (20.2–24.8)	0.80
Number of previous pelvic surgeries (median, 25th–75th percentiles)	0 (0–1)	0 (0–1)	0.92
Menstrual cycle (*N*, %)			0.08
Regular	25 (71.4)	21 (95.5)	
Oligomenorrhea	8 (22.9)	1 (4.5)	
Amenorrhea	2 (5.7)	0 (0)	
History of subfertility (*N*, %)	0 (0)	1 (4.5)	0.39
Gravida at diagnosis (median, 25th–75th percentiles)	2 (2–4)	3 (2–3.3)	0.87
Parity at diagnosis (median, 25th–75th percentiles)	1 (0–2)	1 (0–1)	0.28
Number of previous curettage/suction evacuation (median, 25th–75th percentiles)	0 (0–1)	0.5 (0–1)	0.22
Antecedent pregnancy (*N*, %)			0.13
Miscarriage/abortion/ectopic pregnancy	2 (5.7)	3 (13.6)	
Partial mole	3 (8.6)	0 (0)	
Complete mole	23 (65.7)	18 (81.8)	
Normal pregnancy	7 (20.0)	1 (4.5)	
Pretreatment contraception method (*N*, %)			0.51
None	27 (77.1)	16 (72.7)	
Barrier	7 (20.0)	5 (22.7)	
Combined hormonal contraception	1 (2.9)	0 (0)	
Progestogen only contraception	0 (0)	1 (4.5)	
Intra‐uterine contraceptive device	0 (0)	0 (0)	
Sterilization	0 (0)	0 (0)	
Post‐treatment contraception method (*N*, %)			0.93
None	1 (2.9)	1 (4.5)	
Barrier	32 (91.4)	20 (90.9)	
Combined hormonal contraception	2 (5.7)	1 (4.5)	
Progestogen only contraception	0 (0)	0 (0)	
Intra‐uterine contraceptive device	0 (0)	0 (0)	
Sterilization	0 (0)	0 (0)	
Stage (*N*, %)			0.77
I	28 (80.0)	18 (81.8)	
III	5 (14.3)	2 (9.1)	
IV	2 (5.7)	2 (3.4)	
Risk (*N*, %)			0.18
Low risk	27 (77.1)	20 (90.9)	
High risk	8 (22.9)	2 (9.1)	
WHO score (median, 25–75th percentiles)	3 (1–4)	2 (1–3.5)	0.29
Chemotherapy regimen (*N*, %)			0.33
Single‐agent chemotherapy	21 (60.0)	16 (72.7)	
Combination chemotherapy	14 (40.0)	6 (27.3)	
Number of cycles (*N*, %)			0.34
≤3 cycles	10 (28.6)	9 (40.9)	
>3 cycles	25 (71.4)	13 (59.1)	

**TABLE 4:** Multivariable analysis of different clinical parameters on MoM of serum AMH level in GTN patients 24 months post‐treatment.

Parameters	OR	95% CI	*p*
Age at baseline	0.97	0.87–1.09	0.64
Number of previous curettage/suction evacuation	1.65	0.75–3.62	0.21
World Health Organization score (<7 vs. > =7)	0.26	0.03–2.26	0.22
Chemotherapy regimen (single vs. combination chemotherapy)	1.03	0.22–4.72	0.97
Number of cycles of chemotherapy	0.79	0.22–2.84	0.72

Abbreviations: CI, confidence interval; OR, odds ratio.

### 3.5 Pregnancy outcomes

Finally, after a median follow‐up of 6.9 years (25th–75th percentiles 4.4–8.5 years) we reviewed the subsequent pregnancy outcomes of patients with GTN and the MP group, and found that there was no difference in the pregnancy outcomes (Table 5). Similarly, we compared the pregnancy outcomes between the 16 patients receiving single‐agent and 11 patients receiving combination chemotherapy who attempted pregnancies (Table 6). Although there was a 25.6% difference in the 2‐year accumulative pregnancy rate between the two groups, such difference became less obvious with time, where the 4‐year accumulative pregnancy rate (81.3% vs. 63.6%; *p* = 0.56) and livebirth rate (75.0% vs. 83.3%; *p* = 0.82) were similar. Although the difference was not significant, it was noteworthy that the median time from the completion of chemotherapy leading to the first pregnancy was almost 1 year later in the combination group compared to the single‐agent group (2.88 vs. 1.88 years).

**TABLE 5:** Pregnancy outcomes of patients with GTN and molar pregnancies.

	Control	GTN	*p*
Number of patients attempting pregnancies (*N*, %)	14 (73.7)	27 (47.4)	0.08
Number of patients requiring in‐vitro fertilization	0	2	0.78
Median interval from completion of chemotherapy to first pregnancy (years, 25th–75th percentiles)	NA	2.58 (1.42–3.46)	NA
Accumulative pregnancy rate (*N*, %)			
2‐year	5/14 (35.7%)	9/27 (33.3%)	1.00
3‐year	9/14 (64.3%)	16/27 (59.3%)	1.00
4‐year	10/14 (71.4%)	20/27 (71.4%)	1.00
Total number of pregnancies (*N*, %)	17	38	
Miscarriage	0	4 (10.5)	0.41
Termination of pregnancy	0	2 (5.3)	0.85
Ectopic pregnancy	0	1 (2.6)	1.00
Partial mole	0	1 (2.6)	1.00
Livebirths	17 (100%)	30 (78.9)	0.10

**TABLE 6:** Pregnancy outcomes of patients receiving single‐agent and combination chemotherapy for GTN.

	Single‐agent chemotherapy	Combination chemotherapy	*p*
Number of patients attempting pregnancies (*N*, %)	16 (43.2%)	11 (55.0%)	0.57
Number of patients requiring in‐vitro fertilization	0	2^a^	0.31
Median interval from completion of chemotherapy to first pregnancy (years, (25th–75th percentiles)	2.08 (1.17–3.42)	3.04 (2.35–4.33)	0.10
Accumulative pregnancy rate (*N*, %)			
2‐year	7/16 (43.8)	2/11 (18.2)	0.33
3‐year	11/16 (68.8)	5/11 (45.5)	0.42
4‐year	13/16 (81.3)	7/11 (63.6)	0.56
Total number of pregnancies (*N*, %)	20	18	
Miscarriage	2 (10.0)	2 (11.1)	1.00
Termination of pregnancy	1 (5.0)	1 (5.6)	1.00
Ectopic pregnancy	1 (5.0)	0 (0)	1.00
Partial mole	1 (5.0)	0 (0)	1.00
Livebirths	15 (75.0)	15 (83.3)	0.82
Median number of cycles of chemotherapy for patients with pregnancies (*N*, range)	5 (1–8)	8 (6–13)	<0.001
Median number of cycles of chemotherapy for patients with livebirths (*N*, 25th–75th percentiles)	5 (1–8)	8 (6–13)	0.003

^a^ One patient required in‐vitro fertilization due to male factor.

## 4 DISCUSSION

GTN affects reproductive‐aged women and future fertility is one of the important concerns related to their quality of life. Iwase et al. showed that chemotherapy affected the ovarian reserve in GTN patients compared to patients with MP without GTN.^8^ However, that study was limited by a small sample size containing 22 patients only. In contrast, our study did not show a significant difference in MoM between patients with GTN and MP. And as the choice of chemotherapy and the number of cycles varied among GTN patients, we further analyzed the effects of combination chemotherapy on the change of MoM of serum AMH level and demonstrated a significant drop at 12 months after the cessation of treatment, which then became static. Some patients receiving combination chemotherapy also had a favorable pregnancy outcome in our cohort.

Methotrexate was the most common single agent used for the treatment of GTN in our center. It could impair ovarian reserve by targeting actively proliferating cells, including granulosa cells and oocytes.^14^ While single low‐dose methotrexate used for the treatment of ectopic pregnancies had minimal impact on ovarian reserve,^15^ the effects of higher doses at 3–16 g/m^2^,^16^, ^17^ or repeated doses, of methotrexate had not been well evaluated. Previous studies raised concerns about its gonadotoxicity, as iatrogenic menopause was reported in up to 68% of patients with breast cancers who received high‐dose methotrexate.^18^ However, similar to the finding of the review by Joneborg et al.,^19^ we showed that the MoM of serum AMH in GTN patients receiving methotrexate alone was not significantly compromised.

Combination chemotherapy is generally more gonadotoxic than single‐agent chemotherapy. One retrospective study showed that patients receiving combination EMA‐CO chemotherapy for GTN (median age 49 years, range 25–56 years) had menopause 3 years earlier than those receiving methotrexate alone (median age 51 years, range 25–56 years) (log‐rank chi‐square test = 8.3, *p* = 0.004).^20^ The combination regimens used in our unit were EMA‐CO and CHAMOC. Among these medications, cyclophosphamide was thought to have the highest gonadotoxic potential. One article that studied 17 breast cancer or lymphoma patients (aged 19–35 years) showed that their serum AMH levels dropped to a low or undetectable level after 1–2 cycles of multi‐agent chemotherapy, and the median level of AMH for those receiving alkylating agents remained to be lower than 0.05 ng/mL (range 0.05–0.54 ng/mL) 1 year after treatment.^21^ Etoposide was also shown to be associated with reduced serum AMH levels, which might be related to its inhibition of DNA synthesis and DNA repair by forming complexes with topoisomerase II and DNA.^8^ Another study showed that 50% of patients using etoposide for GTN had raised basal serum luteinizing hormone (LH) and follicle‐stimulating hormone (FSH) in an age‐dependent manner, where ovarian function resumed within 121 days after the cessation of treatment for those younger than 39 years.^22^

Our results showed that despite the decreasing trend in the MoM from the baseline in patients receiving combination chemotherapy, the MoM level actually became static after 12 months. Besides, multivariable analysis showed that the use of combination chemotherapy was not a significant parameter in determining the MoM level. Furthermore, despite using a median of 8 cycles of combination chemotherapy, the livebirth rate in this group was not inferior to that in patients receiving single‐agent chemotherapy. These results implied that ovarian function might improve with time following chemotherapy. Nonetheless, the 2‐year pregnancy rate was 25.6% lower and the median time from completion of chemotherapy to the first pregnancy was longer in patients receiving combination chemotherapy compared to those receiving single‐agent chemotherapy. This highlights the importance of fertility counseling for patients requiring combination chemotherapy, especially those who wish to contemplate pregnancy early. In the presence of other risk factors for infertility such as age, fertility preservation treatments, such as oocyte or embryo cryopreservation,^23^ should be considered. On the other hand, immune checkpoint inhibitors (ICIs) have recently been proven to be effective in treating advanced and recurrent GTN and successful pregnancy had been reported.^24^ Some suggested that ICIs could be considered as an alternative treatment instead of high‐dose, prolonged, or combination chemotherapy.^19^ However, the experience is still limited due to the rarity of the disease, and careful counseling and monitoring are required.^25^

AMH is a glycoprotein synthesized almost exclusively synthesized by the granulosa cells in the pre‐antral and small antral ovarian follicles in adult women, and so its level can reflect the primordial follicle pool size. Its level is static throughout the menstrual cycle and has low inter‐cycle variability.^26^ Therefore, it has been widely used as a surrogate measure of the primordial follicle pool. The measurement is also simpler to perform and less labour‐intensive than transvaginal ultrasound measurement of antral follicle count. Earlier studies revealed a relationship between AMH and spontaneous fertility or fecundity,^27^ and its level could predict a decline in reproductive capacity in healthy women.^28^ Patients with a lower AMH level were also found to have a poorer response and prognosis in assisted reproductive technologies.^29^

Nevertheless, the association between AMH and natural conception rate was still controversial.^30^, ^31^ In GTN, the predictive value of AMH in post‐chemotherapy residual fertility is even more unclear. First, the effect of AMH can be age‐dependent.^31^ Second, some have shown that the use of an absolute AMH level alone for clinical decision‐making could be misleading.^29^ Therefore, using MoM might avoid the influence of progressive age on the AMH level during the longitudinal follow‐up.^12^ Our study was the first longitudinal study that utilized MoM of serum AMH levels in patients who underwent chemotherapy for GTN. We demonstrated a decreasing trend of MoM in those receiving combination chemotherapy, but the drop became static between 12 and 24 months. Due to the retrospective nature of this study where a certain proportion of patients did not have archived blood beyond 24 months, the change of MoM of serum AMH beyond 24 months and the correlation with pregnancy outcomes could not be evaluated in the current study.

One study showed that higher pretreatment AMH levels were predictive of recovery of ovarian function after treatment, independent of patients' age and type of chemotherapy.^21^ We suggest that AMH should be incorporated as part of the pretreatment workup algorithm for GTN where the MoM of serum AMH can be calculated. Patients with a low value should be referred to reproductive medicine specialists for a proper assessment and to discuss fertility preservation issues. Lastly, it is noteworthy that only around half of the patients receiving chemotherapy attempted pregnancy. The psychological burden of GTN was not addressed in this study. Multi‐disciplinary care with support from nurses and psychologists should also be integrated into the management plan.

## 5 CONCLUSION

We showed that GTN did not adversely affect the MoM compared to the MP, especially those who received single‐agent chemotherapy only. In contrast, patients using combination chemotherapy had a significant drop of MoM at 12 months after cessation of treatment, but it became static at 24 months. Pregnancy was feasible despite the use of multiple courses of combination chemotherapy. Our results provided further information on the ovarian reserve and pregnancy outcomes in this rare disease and could be useful in counseling GTN patients who wish to contemplate pregnancy. Prompt referral to reproductive medicine specialists is needed for patients requiring combination chemotherapy, especially those wish to start pregnancy 1–2 years after completion of treatment, or those with other risk factors like age where early intervention may have to be considered.

## AUTHOR CONTRIBUTIONS

**Theodora Hei Tung Lai:** Data curation (equal); formal analysis (equal); funding acquisition (equal); project administration (equal); writing – original draft (equal); writing – review and editing (equal). **Lesley Suk Kwan Lau:** Data curation (equal); formal analysis (equal); methodology (equal). **Siew‐Fei Ngu:** Writing – review and editing (equal). **Mandy MY Chu:** Writing – review and editing (equal). **Karen KL Chan:** Writing – review and editing (equal). **Ernest Hung Yu Ng:** Writing – review and editing (equal). **Raymond Hang Wun Li:** Formal analysis (equal); methodology (equal); supervision (equal); writing – review and editing (equal). **Ka Yu Tse:** Conceptualization (lead); data curation (equal); formal analysis (equal); funding acquisition (equal); methodology (equal); project administration (lead); supervision (equal); writing – original draft (equal); writing – review and editing (lead).

## FUNDING INFORMATION

This research was funded by the Professor PC Ho Research and Development Fund in Reproductive Medicine, The University of Hong Kong.

## CONFLICT OF INTEREST STATEMENT

The authors declare no conflict of interest. The funders had no role in the design of the study; in the collection, analyses, or interpretation of data; in the writing of the manuscript; or in the decision to publish the results.

## CONSENT

Informed consent was obtained from all subjects involved in the study.

## INSTITUTIONAL REVIEW BOARD STATEMENT

The study was approved by the Institutional Review Board of The University of Hong Kong/Hospital Authority Hong Kong West Cluster (IRB number: UW 11‐298).

## Supporting information


Table S1.

Table S2.

## Data Availability

All data generated in the current study were included in the manuscript. Other raw data are available from the corresponding author upon reasonable request.
