# Supplementary material for: Comparison of the multiples of the median of serum anti‐müllerian hormone and pregnancy outcomes in patients with gestational trophoblastic disease: A case–control study
Source: Cancer Med. 2024 Mar 28;13(7):e7134. doi: 10.1002/cam4.7134 (PMC10973878; doi:10.1002/cam4.7134)
Supplement: Supplementary file 1 — Table S1. [file CAM4-13-e7134-s001.docx]

| Comparison | Molar pregnancy | | GTN | |
| --- | --- | --- | --- | --- |
|  | Z | P value | Z | P value |
| Month 6 vs baseline | -1.01 | 0.315 | -1.27 | 0.204 |
| Month 12 vs baseline | -1.13 | 0.258 | -2.79 | 0.005 |
| Month 24 vs baseline | -0.63 | 0.53 | -0.73 | 0.468 |

**Supplementary Table 1** Comparison of MoM of AMH from baseline within individual group in patients with molar pregnancy and GTN
