# Supplementary material for: Comparison of the multiples of the median of serum anti‐müllerian hormone and pregnancy outcomes in patients with gestational trophoblastic disease: A case–control study
Source: Cancer Med. 2024 Mar 28;13(7):e7134. doi: 10.1002/cam4.7134 (PMC10973878; doi:10.1002/cam4.7134)
Supplement: Supplementary file 2 — Table S2. [file CAM4-13-e7134-s002.docx]

| Comparison | Single-agent chemotherapy | | Combination chemotherapy | |
| --- | --- | --- | --- | --- |
|  | Z | P value | Z | P value |
| Month 6 vs baseline | -0.63 | 0.528 | -1.29 | 0.198 |
| Month 12 vs baseline | -1.49 | 0.137 | -2.69 | 0.007 |
| Month 24 vs baseline | -0.5 | 0.62 | -1.9 | 0.058 |

**Supplementary Table 2** Comparison of MoM of AMH from baseline within individual group in patients with single-agent and combination chemotherapy
